# Viral Load and Crimean-Congo Hemorrhagic Fever

**DOI:** 10.3201/eid1305.061588

**Published:** 2007-05

**Authors:** Anna Papa, Christian Drosten, Silvia Bino, Εvangelia Papadimitriou, Marcus Panning, Enkelejda Velo, Majlinda Kota, Arjan Harxhi, Antonis Antoniadis

**Affiliations:** * Aristotle University of Thessaloniki School of Medicine, Thessaloniki, Greece; † Berhard Nocht Institute for Tropical Medicine, Hamburg, Germany; ‡ Institute of Public Health, Tirana, Albania; § University Hospital, Tirana, Albania

**Keywords:** Crimean-Congo hemorrhagic fever, viral load, Albania, letter

**To the Editor:** Crimean-Congo hemorrhagic fever (CCHF) is a severe viral disease transmitted to humans by tick bite or contact with blood, excreta, or tissues of infected patients or livestock. The disease is endemic in many African, Asian, and European countries. Sporadic cases or outbreaks have been observed in the Balkan Peninsula (*1*–*5*). Prompt diagnosis of the disease is essential for preventing human-to-human transmission. Reverse transcription–PCR (RT-PCR) is the detection method of choice in first days of illness and in severe cases with no antibody production. In recent years, real-time RT-PCR approaches have been described for detection and quantification of CCHF virus (*6*–*8*). However, no information is available on viral RNA concentration in patients. We describe a real-time RT-PCR for detection and quantification of CCHF virus, present the results of its use with clinical samples, and report the relationship between viral load and severity and outcome of CCHF.

We tested 29 serum samples from Albanian patients with suspected CCHF or their contacts who were living in a CCHF-endemic area of Albania. Serum samples were collected during 2003–2006 and categorized into 3 groups. Group A contained samples from 11 patients with CCHF confirmed by a conventional RT-nested PCR (*9*). Group B contained samples from 5 patients who had negative RT-nested PCR results and positive serologic results. Group C contained samples from 15 persons who were from the same region as the CCHF patients but who did not have any clinical symptoms of CCHF and had negative PCR or serologic results.

One set of primers and 1 probe were designed to amplify an 84-bp genome region of the S RNA segment of CCHF virus on the basis of European sequences (Balkan and Russian strains available in GenBank): primers CCEuS 5′-TGACAGCATTTCTTTAACAGACATCA-3′ and CCEuAs 5′-AAACACGGCAGCCTTAAGCA-3′, and probe 5′-TCGCCAGGGACTTTATATTCTGCAAGG-3′. A 25-μL reaction was conducted in a LightCycler (Roche, Indianapolis, IN, USA) with 10 mmol/L of each deoxynucleotide triphosphate, 600 nmol/L of each primer, 200 nmol/L of probe, and 3 μL of RNA. Cycling conditions were 50°C for 30 min and 95°C for 15 min, followed by 45 cycles at 95°C for 15 s and 58°C for 30 s. A quantification curve was constructed with 10-fold serial dilutions of in vitro–transcribed CCHF virus RNA. Positive results were obtained up to a dilution of 10^−12^, which corresponds to ≈45 virus genome equivalents (geqs) per reaction.

Twelve samples had positive results: all 11 samples in group A and 1 in group B (Table). Results for the remaining samples in groups B and C were negative. Levels of tumor necrosis factor-α (TNF-α), interleukin-6 (IL-6), IL-10, and a 60-kDa soluble receptor of TNF were previously measured in most of the samples in this study (*10*), and their values are shown in the Table.

**Table:** Epidemiologic, molecular, and clinical data for 12 Albanian patients with suspected Crimean-Congo hemorrhagic fever, 2003–2006*

Patient	Day of illness	In hospital	Outcome	Real-time RT-PCR, geqs/reaction	IFA	TNF-α, pg/mL	sTNF-R, ng/mL	IL-6, pg/mL	IL-10, pg/mL	Leukocytes, × 10^9^/L	Platelets, × 10^9^/L
Group A primary											
23/03	6	Yes	D	28,990,000	+	68.5	14.0	109.7	388.3	1,700	36,200
82/03	5	Yes	R	450	+	N	N	17.0	23.9	2,300	96,830
154/04	18	Yes	R	33	+	ND	ND	ND	ND	15,000	71,400
178/04	4	Yes	R	7,271,000	+		ND	ND	ND	4,100	62,550
252/06	2	Yes	R	4049	–	ND	ND	ND	ND	3,700	117,900
Group A secondary											
24b/03	9	Yes	R	166	+	1,444.8	N	114.2	N	4,800	63,800
25b/03	9	Yes	R	40	+	N	N	10.3	N	3,800	63,000
50/03	3	No	R	240	–	N	N	N	43.4	ND	ND
52/03	3	No	R	18	–	N	N	N	9.9	ND	ND
56/03	5	No	R	62	+	N	N	26.1	N	ND	ND
34/03	5	Yes	R	46	–	N	8.9	N	N	8,000	102,000
Group B secondary											
40/03	7	No	R	14	+	N	N	N	23.1	ND	ND

*RT-PCR, reverse transcription–PCR; geqs, genome equivalents; IFA, immunofluorescent assay; TNF-α, tumor necrosis factor-α; sTNF R, soluble TNF-α receptor; IL-6, interleukin-6; D, died; +, positive; R, recovered; N, normal value; ND, not done; –, negative.

Viral loads ranged from 14 × 10^6^ to 28.99 × 10^6^ geqs/reaction. The highest level was observed in the patient who died (23/03). High loads were observed in all primary case-patients (23/03, 82/03, 178/04, 252/06) except for patient 154/04, from whom a sample was obtained 18 days after onset of disease. All primary case-patients had severe disease with high fever and clinically apparent hemorrhage. All secondary case-patients, except patient 34/03, were contacts of the patient who died (24b/03, husband; 25b/03, brother-in-law; 50/03 and 52/03, cousins; 56/03, sister-in-law; 40/03, son of sister) and had symptoms of disease ≈1 week after the death of patient 23/03.

Viral load of secondary case-patients was <250 geqs/reaction, which was much lower than that of primary case-patients. This finding suggests that the disease is more severe in primary case-patients and becomes a milder form in secondary case-patients. Samples of secondary case-patients 24b/03 and 25b/03 were obtained on day 9 of illness, and patient 24b/03 had a 4× higher viral load than patient 25b/03. A possible explanation might be that because patient 24b/03 had closer contact with the person who died, he received a higher dose of virus, which might affect severity of the disease. Other secondary case-patients had milder symptoms with no clinically apparent hemorrhage and were not hospitalized. All hospitalized patients had leukopenia, except for the patient whose sample was taken 18 days after the onset of disease. No correlation was observed between viral load and cytokine levels or platelet counts, which suggests that other factors are involved in pathogenicity and immune response.

The real-time RT-PCR was rapid and more sensitive than the RT-nested PCR because 1 additional positive sample was detected. Samples with positive results from the first round of the conventional RT-nested PCR (23/03, 178/04, 252/06) had the highest viral loads when tested by real-time RT-PCR.

In conclusion, a 1-step real-time RT-PCR for detection and quantification of CCHF virus was developed, used with clinical samples, and provided informative data on the severity, course, and outcome of CCHF. Further studies, preferably in serial samples of patients, should provide insights into the pathology of CCHF and the effectiveness of antiviral drugs.

## References

[1] Papa A, Bino S, Llagami A, Brahimaj B, Papadimitriou E, Pavlidou V, Crimean-Congo hemorrhagic fever in Albania, 2001. Eur J Clin Microbiol Infect Dis. 2002;21:603–6. 10.1007/s10096-002-0770-9 12226691

[2] Papa A, Bozovic B, Pavlidou V, Papadimitriou E, Pelemis M, Antoniadis A. Genetic detection and isolation of Crimean-Congo hemorrhagic fever virus, Kosovo, Yugoslavia. Emerg Infect Dis. 2002;8:852–4. 12141973 10.3201/eid0808.010448 PMC2732509

[3] Papa A, Christova I, Papadimitriou E, Antoniadis A. Crimean-Congo hemorrhagic fever in Bulgaria. Emerg Infect Dis. 2004;10:1465–7. 15496250 10.3201/eid1008.040162 PMC3320408

[4] Ahmeti S, Raka L. Crimean-Congo haemorrhagic fever in Kosova: a fatal case report. Virol J. 2006;3:85. 10.1186/1743-422X-3-85 17034648 PMC1624825

[5] Drosten C, Minnak D, Emmerich P, Schmitz H, Reinicke T. Crimean-Congo hemorrhagic fever in Kosovo. J Clin Microbiol. 2002;40:1122–3. 10.1128/JCM.40.3.1122-1123.2002 11880460 PMC120293

[6] Drosten C, Gottig S, Schilling S, Asper M, Panning M, Schmitz H, Rapid detection and quantification of RNA of Ebola and Marburg viruses, Lassa virus, Crimean-Congo hemorrhagic fever virus, Rift Valley fever virus, dengue virus, and yellow fever virus by real-time reverse transcription-PCR. J Clin Microbiol. 2002;40:2323–30. 10.1128/JCM.40.7.2323-2330.2002 12089242 PMC120575

[7] Duh D, Saksida A, Petrovec M, Dedushaj I, Avsic-Zupanc T. Novel one-step real-time RT-PCR assay for rapid and specific diagnosis of Crimean-Congo hemorrhagic fever encountered in the Balkans. J Virol Methods. 2006;133:175–9. 10.1016/j.jviromet.2005.11.006 16343650

[8] Yapar M, Aydogan H, Pahsa A, Besirbellioglu A, Bodur H, Basustaoglu AC, Rapid and quantitative detection of Crimean-Congo hemorrhagic fever virus by one-step real-time reverse transcriptase-PCR. Jpn J Infect Dis. 2005;58:358–62. 16377867

[9] Schwarz TF, Nsanze H, Longson M, Nitschko H, Gilch S, Shurie H, Polymerase chain reaction for diagnosis and identification of distinct variants of Crimean-Congo hemorrhagic fever virus in the United Arab Emirates. Am J Trop Med Hyg. 1996;55:190–6. 8780459 10.4269/ajtmh.1996.55.190

[10] Papa A, Bino S, Velo E, Harxhi A, Kota M, Antoniadis A. Cytokine levels in Crimean-Congo Hemorrhagic Fever. J Clin Virol. 2006;36:272–6. 10.1016/j.jcv.2006.04.007 16765637

